# Study of Heat and Mass Transfer in MHD Flow of Micropolar Fluid over a Curved Stretching Sheet

**DOI:** 10.1038/s41598-020-61439-8

**Published:** 2020-03-12

**Authors:** Asia Yasmin, Kashif Ali, Muhammad Ashraf

**Affiliations:** 1 Center for Advanced Studies in pure and Applied Mathematics, Bahauddin Zakariya University Multan, Multan, Pakistan; 2 Department of Basic Sciences and Humanities, Muhammad Nawaz Sharif University of Engineering and Technology, Multan, Pakistan

**Keywords:** Environmental impact, Applied mathematics

## Abstract

A comprehensive investigation of mass and heat transfer in magnetohydrodynamics (MHD) flow of an electrically conducting non-Newtonian micropolar fluid because of curved stretching sheet is presented. Flow is originated by stretching of curved sheet by means of linear velocity. Concentration and energy equations are incorporated to study repercussion of mass and heat transfer. To define basic equations of the model, curvilinear coordinates are used. The transformed BL (boundary layer) equations for the momentum, concentration, angular momentum and temperature with appropriate boundary conditions are numerically solved by SOR (successive over relaxation) algorithms combined with the quasi-linearization technique. Flow features such as temperature fields, micro rotation, velocity and concentration are appraised for manipulation of pertinent parameters. The radius of curvature enhances the temperature and concentration whereas it declines micro-rotation as well as velocities of the fluid. It is significant to notice that magnetic field interaction is caused counterproductive in increasing concentration distribution and fluid temperature while diminishing micro-rotation and velocities at all domain flow points. As schmidt number increases concentration of fluid reduces.

## Introduction

Flow of mass and heat transfer due to surface stretching is important and has noteworthy interest due to its enormous applications in industry and engineering. We are mentioning only a few utilizations like expulsion of plastic & rubber sheets, metal revolve, crystal growing, drawing of plastic films and paper production. The exact solution in closed form of fluid past a stretching sheet was presented first time by Crane^1^. Many authors such as Gupta^2^, Char and Chen^3^, Dutta *et al*.^4^ and Bhattacharyya *et al*.^5^ expanded Crane^1^ study for Newtonian as well as non-Newtonian fluids by including impacts of heat and mass transport under diverse situations. Heat transfer and flow in boundary layer on permanently moving surfaces was evaluated by Tsou *et al*.^6^. Bhukta *et al*.^7^ deliberated heat transfer and mass effect on an electrically carrying out viscoelastic fluid of shrinking sheet in existence of heat source. The precise solution for temperature field was attained and applied magnetic field enhanced the velocity and concentration distribution.

The curved sheets or flat stretching plates in case of heat transfer and boundary layer flow are of pragmatic importance in extrusion processes, fiber technology as well as in analytical interest. Basically, plastic films and polymer sheets manufacturing depends on that technology. A lot of examples incorporate the cooling bath for infinite metallic plate cooling, boundary layer in condensation technique of liquid film, glass blowing, polymer extrusion, paper production, plastic films drawing and metal spinning. Final product quality is based on the heat transfer rate at stretching surface.

Naveed *et al*.^8^ examined MHD BL (boundary layer) unsteady flow above curved stretching surface. Abbas *et al*.^9^ examined numerically radiation impacts on MHD flow above curved stretching surface of nanofluid by assimilating the slip, collective radiation and heat generation effects. Sahoo^10^ investigated the mass and heat transfer in MHD flow of viscoelastic fluid via porous media bounded by vacillating plate in slip flow system. Singh *et al*.^11^ have inspected mass transfer and heat in MHD flow or viscous fluid past a straight up plate in oscillatory velocity suction.

Mittal and Kataria^12^ applied homotopy analysis technique to study three dimensional nanofluid flow of CuOe water via porous media between two horizontal plates which were placed parallel. Kataria and Mittal^13^ analyzed analytical interpretation of unsteady hydromagnetic flow in presence of transverse uniform magnetic field and thermal radiation of optically thick nanofluid past on oscillating vertical plate. Mathematical modeling of heat and mass transfer of unsteady electrically conducting MHD nanofluid past on vertically oscillating plate was considered by Kataria and Mittal^14^. Nonlinear stretched surface of micropolar flow of ferrofluid was described by Mittal *et al*.^15^ while involving vital imperative phenomena’s such as joule heating, viscous dissipation and mixed convection. Mittal^16^ scrutinized mass and heat transfer in three dimensional composites of water based MHD nanofluid (nanoparticles of Al_2_O_3_ and CuO) flow via parallel horizontal plates in rotating system. Magnetic field influence on squeezing nanofluid between two equidistance plates has been studied by Mittal^17^. Mittal and Patel^18^ considered Brownian motion, heat generation, thermal radiation, chemical reaction and thermophoresis in two dimensional MHD mixed convective stagnation Casson fluid flow over infinite plate embedded in a porous media.

Zhixiong *et al*.^19^ employed technique of lattice Boltzmann to investigate the influence of Darcy, magnetic and Reynolds numbers on water based nanofluid flow through a permeable duct. Microstructural and inertial features influence on MHD micropolar nanofluid had been inspected by Patel *et al*.^20^ by utilizing similarity transformation. In magnetic field presence, Sheikholeslami *et al*.^21^ has studied heat transfer in Al_2_O_3_-water nanofluid flow features through porous media between parallel horizontal plates. Sheikholeslami *et al*.^22^ presented heat generation and thermal diffusion impacts on unsteady MHD radiating nanofluid past an oscillating plate via porous media. Kataria and Mittal^23^ scrutinized the Casson nanofluid flow over vertically oscillating plate along isothermal wall temperature taking the impacts of radiation and magnetic field. MHD nanofluid flow between two parallel horizontal plates in a porous media had been explained by Kataria and Mittal^24^ while considering impacts of Brownian motion, volume fraction, interfacial thermal resistance and nanoparticles size.

Researchers namely Machireddy and Naramgari^25^, Rashidi *et al*.^26^ and Chandrasekar and Kasiviswanathan^27^ studied transfer of heat and mass properties of Newtonian and non Newtonian fluid flows in existence of applied magnetic field. Rashidi *et al*.^28^ studied two dimensional steady MHD viscoelastic fluid flow above straight up stretching surface in occurrence of Soret and Dufour effects systematically via HAM. Dabe *et al*.^29^ examined numerical results of MHD flow with mass and heat transfer in a fluid coming towards stagnation position on upright plate. In that study, they considered both weak concentrations(*n* = 1/2) and strong concentrations (*n* = 0). Hayaat *et al*.^30^ investigated convective mass and heat transfer effects for Eyring-Powell fluid past a leaning exponential stretching surface. Imtiaz *et al*.^31^ analyzed Jeffrey fluid owed to stretching curved sheet. Outcome of heterogeneous and homogenous reactions was taken into account. Singh^32^ analyzed the impact of buoyancy and magnetic field on heat transfer flow among alumina water nanofluid surroundings and moving sheet.

The mathematical reproduction in case of stretching curved sheet along regular curvature was firstly dispensed by Saajid *et al*.^33^. They retrieved equations by using curvilinear coordinates system. Abbas *et al*.^34^ considered heat and flow exchange of fluid which is electrically conducting for stretching curved surface with stable and changeable surface temperature. Naveed *et al*.^35^ discussed influence of magnetic field and radition on micropolar fluid past over a stretching curved surface. Rosca and Pop^36^ investigated boundary layer unsteady passed over a curved extending/shrinking surface.

The main intention of this work is to determine the impacts of mass and heat transfer in magnetohydrodynamics (MHD) flow of an electrically conducting non-Newtonian micropolar fluid due to a curved stretching sheet. Many researchers were reported curved stretching sheet, a few researchers were scrutinized about magnetohydrodynamics (MHD) flow on curved sheet. So due to this rather than other researchers we used MHD flow along heat and concentration parameter. Numerical solution for fluid velocity, temperature, microrotation and meditation distribution is acquired by means of SOR algorithm. Results are presented via graphs.

## Problem formulation

The physical configuration consists BL micropolar fluid with mass and heat transfer over curved stretching sheet under magnetic field influence. The flow is two dimensional, incompressible and steady. Let *u* & v be velocity components in *s*_1_*-* and *r*_1_-directions, respectively. After using BL approximations the motion equations for present study are

1 $$\frac{\partial }{\partial {r}_{1}}\{({r}_{1}+{R}_{1})v\}+{R}_{1}\frac{\partial u}{\partial {s}_{1}}=0$$

2 $$\frac{{u}^{2}}{{r}_{1}+{R}_{1}}=\frac{1}{\rho }\,\frac{\partial p}{\partial {r}_{1}}$$

3 $$\{\begin{array}{c}v\frac{\partial u}{\partial {r}_{1}}+\,\frac{{R}_{1}\,u}{{r}_{1}+{R}_{1}}\,\frac{\partial u}{\partial {s}_{1}}+\frac{u\,v}{{r}_{1}+{R}_{1}}=-\,\frac{1}{\rho }\,\frac{{R}_{1}}{{r}_{1}+{R}_{1}}\,\frac{\partial p}{\partial {s}_{1}}+\left(\upsilon +\frac{\kappa }{\rho }\right)\\ \left(\frac{{\partial }^{2}u}{\partial {{r}_{1}}^{2}}+\frac{1}{{r}_{1}+{R}_{1}}\frac{\partial u}{\partial {r}_{1}}-\frac{u}{{({r}_{1}+{R}_{1})}^{2}}\right)-\frac{\kappa }{\rho }\frac{\partial L}{\partial {r}_{1}}-\frac{\sigma {B}_{0}^{2}}{\rho }\,u\end{array}$$

4 $$\{\begin{array}{c}v\,\frac{\partial L}{\partial {r}_{1}}+\frac{{R}_{1}\,u}{{r}_{1}+{R}_{1}}\,\frac{\partial L}{\partial {s}_{1}}=\frac{\gamma }{\rho \,j}\left(\frac{{\partial }^{2}L}{\partial \,{{r}_{1}}^{2}}+\frac{1}{{r}_{1}\,+\,{R}_{1}}\,\frac{\partial \,L}{\partial \,{r}_{1}}\right)\\ -\frac{\kappa }{\rho \,j}\left(2\,L+\frac{\partial \,u}{\partial \,{r}_{1}}+\frac{u}{{r}_{1}+\,{R}_{1}}\right)\end{array}$$

5 $$\rho \,{c}_{p}\left[v\frac{\partial T}{\partial {r}_{1}}\,+\frac{u\,{R}_{1}}{{r}_{1}+{R}_{1}}\frac{\partial T}{\partial {s}_{1}}\right]={k}_{1}\left[\frac{{\partial }^{2}T}{\partial \,{r}_{1}}+\frac{1}{{r}_{1}+{R}_{1}}\,\frac{\partial \,T}{\partial \,{r}_{1}}\right]$$

6 $$v\frac{\partial C}{\partial {r}_{1}}+\frac{u\,{R}_{1}}{{r}_{1}+{R}_{1}}\frac{\partial C}{\partial {s}_{1}}=D\left[\frac{{\partial }^{2}C}{\partial \,{r}_{1}}+\frac{1}{{r}_{1}+{R}_{1}}\,\frac{\partial \,C}{\partial \,{r}_{1}}\right].$$

Where the fluid density is *ρ*, pressure is *p*, micro-rotation is *L* in the *r*_1_ *s*_1_-plane, micro-inertia for each unit mass is *j*, spin gradient viscosity is *γ*, vortex viscosity is $\kappa$, heat capacity is *c*_*p*_, thermal conductivity is *k*_1_, mass diffusion coefficient is *D* and temperature is *T*. Sajid *et al*.^33^ assumed that the pressure is not constant inside BL for curved stretching surface. Nazar *et al*.^37^ and Rees^38^ defined *γ* as

7 $$\gamma =\left(\mu +\frac{\kappa }{2}\right)j,$$

where $j=\upsilon /a{s}_{1}$ is the reference length.

The suitable boundary setting for flow, microrotation, concentration and temperature is

8 $$\{\begin{array}{ccccc}u=a\,{s}_{1}, & v=0, & L=-\,{m}_{0}\frac{{\rm{\partial }}u}{{\rm{\partial }}{r}_{1}},\,\,C={C}_{w},\,\,T={T}_{w} & {\rm{a}}{\rm{t}} & {r}_{1}=0,\\ u\to 0, & \frac{{\rm{\partial }}u}{{\rm{\partial }}{r}_{1}}\to 0, & L\to 0,\,C\to {C}_{{\rm{\infty }}},\,T\to {T}_{{\rm{\infty }}} & {\rm{a}}{\rm{s}} & {r}_{1}\to {\rm{\infty }}\end{array}$$

whereas to reduce the flow, microrotation, temperature and concentration equations into ODEs, we illustrate following new variables

9 $$\{\begin{array}{c}u=a\,{s}_{1}\,f{\rm{{\prime} }}(\eta ),\,v=\frac{-{R}_{1}}{{r}_{1}+{R}_{1}}\,\sqrt{a\,\upsilon }f(\eta ),\,L=a\,s{}_{1}\,\sqrt{\frac{a}{\upsilon }}g(\eta ),\,k=\sqrt{\frac{a}{\upsilon }}{R}_{1}\\ \eta =\sqrt{\frac{a}{\upsilon }\,}\,{r}_{1},\,p=\rho \,{a}^{2}\,{{s}_{1}}^{2}\,P(\eta ),\,\theta (\eta )=\frac{T-{T}_{{\rm{\infty }}}}{{T}_{w}-{T}_{{\rm{\infty }}}},\,\phi (\eta )=\frac{C-{C}_{{\rm{\infty }}}}{{C}_{w}-{C}_{{\rm{\infty }}}}.\end{array}$$

Utilizing Eq. (9), in the conservation Eq. (1) we see that velocity field is well-suited with this Eq. while Eqs. (2–6) may be decreased as:

10 $$\frac{\partial P}{\partial \eta }=\frac{{(f{\prime} )}^{2}}{\eta +k}$$

11 $$\{\begin{array}{c}\frac{2k}{\eta +k}P(\eta )=(1+{C}_{1})(f{\rm{{\prime} }}{\rm{{\prime} }}{\rm{{\prime} }}+\frac{f{\rm{{\prime} }}{\rm{{\prime} }}}{\eta +k}-\frac{f{\rm{{\prime} }}}{{(\eta +k)}^{2}})-{C}_{1}\,g{\rm{{\prime} }}\\ -{M}^{2}f{\rm{{\prime} }}-\frac{k}{\eta +k}({(f{\rm{{\prime} }})}^{2}-f\,f{\rm{{\prime} }}{\rm{{\prime} }}-\frac{f{\rm{{\prime} }}\,f}{\eta +k})\end{array}$$

12 $$\{\begin{array}{c}\left(1+\frac{{C}_{1}}{2}\right)\left(g{\prime\prime} +\frac{1}{\eta +k}g{\prime} \right)+\frac{k}{\eta +k}f\,g{\prime} \\ -\frac{k}{\eta +k}f{\prime} \,g-{C}_{1}\left(2\,g+f{\prime\prime} +\frac{f{\prime} }{\eta +k}\right)=0\end{array}$$

13 $$\theta {\prime\prime} +\frac{1}{\eta +k}(1+k\,Pr\,f)\theta {\prime} =0$$

14 $$\frac{1}{Sc}\,\phi {\prime\prime} +\frac{1}{\eta +k}\left(\frac{1}{Sc}+k\,f\right)\phi {\prime} =0$$

where ${M}^{2}=\frac{\sigma {B}_{0}^{2}}{\rho a}$, $Sc=\frac{\upsilon }{D}$, $\Pr =\frac{\mu \,{c}_{p}}{{k}_{1}}$, $\frac{\kappa }{\mu }={C}_{1}$, $k={R}_{1}\sqrt{a/\upsilon }$ are magnetic parameter, Schmidt number, Prandtl number, material parameter, and radius of curvature respectively.

After getting rid of pressure term from Eqs. (10) and (11) we have

15 $$\{\begin{array}{c}(1+{C}_{1})\left[{f}^{(iv)}+\frac{2\,f\prime\prime\prime }{\eta +k}-\frac{f{\prime\prime} }{{(\eta +k)}^{2}}+\frac{f{\prime} }{{(\eta +k)}^{3}}\right]-\frac{k}{\eta +k}(f{\prime} \,f{\prime\prime} -f\,f\prime\prime\prime )\\ -\frac{k}{{(\eta +k)}^{2}}({(f{\prime} )}^{2}-f\,f{\prime\prime} )-\frac{k}{{(\eta +k)}^{3}}f\,f{\prime} \\ -{C}_{1}\left(g{\prime\prime} +\frac{g{\prime} }{\eta +k}\right)-{M}^{2}\left(\frac{f{\prime} }{\eta +k}+f{\prime\prime} \right)=0\end{array}$$

The corresponding boundary conditions are

16 $$\{\begin{array}{l}f(0)=0,\,f{\prime} (0)=1,\,g(0)=0,\,\theta (0)=1,\,\phi (0)=1\\ f{\prime} (\infty )=0,\,f{\prime\prime} (\infty )=1,\,g(\infty )=0,\,\theta (\infty )=0,\,\phi (\infty )=0\end{array}$$

## Numerical Solution

Quasi-linearization is utilized to build sequences $\{{g}^{(k)}\}$, $\{{\theta }^{(k)}\}$, $\{{\phi }^{(k)}\}$ and $\{{f}^{(k)}\}$ for, micro-rotation, temperature, concentration and velocity distributions that converge to solutions of Eqs. (12–15). For producing $\{{f}^{(k)}\}$, linearized form of Eq. (15) may be written as:

17 $$\{\begin{array}{c}Let:\,\varphi (f,\,{f}_{\eta },\,{f}_{\eta \eta },\,{f}_{\eta \eta \eta },\,{f}_{\eta \eta \eta \eta })\equiv (1+{C}_{1})\left[{f}_{\eta \eta \eta \eta }+\frac{2\,{f}_{\eta \eta \eta }}{\eta +k}-\frac{{f}_{\eta \eta }}{{(\eta +k)}^{2}}+\frac{{f}_{\eta }}{{(\eta +k)}^{3}}\right]\\ -\frac{k}{\eta +k}({f}_{\eta }\,{f}_{\eta \eta }-f\,{f}_{\eta \eta \eta })-\frac{k}{{(\eta +k)}^{2}}({({f}_{\eta })}^{2}-f\,{f}_{\eta \eta })-\frac{k}{{(\eta +k)}^{3}}f\,{f}_{\eta }\\ -{C}_{1}\left({g}_{\eta \eta }+\frac{{g}_{\eta }}{\eta +k}\right)-{M}^{2}\left(\frac{{f}_{\eta }}{\eta +k}+{f}_{\eta \eta }\right),\\ \varphi ({f}^{(k)},\,{f}_{\eta }^{(k)},\,{f}_{\eta \eta }^{(k)},\,{f}_{\eta \eta \eta }^{(k)},\,{f}_{\eta \eta \eta \eta }^{(k)})+({f}^{(k+1)}-{f}^{(k)})\frac{\partial \varphi }{\partial {f}^{(k)}}+({f}_{\eta }^{(k+1)}-{f}_{\eta }^{(k)})\frac{\partial \varphi }{\partial {f}_{\eta }^{(k)}}\\ +({f}_{\eta \eta }^{(k+1)}-{f}_{\eta \eta }^{(k)})\frac{\partial \varphi }{\partial {f}_{\eta \eta }^{(k)}}+({f}_{\eta \eta \eta }^{(k+1)}-{f}_{\eta \eta \eta }^{(k)})\frac{\partial \varphi }{\partial {f}_{\eta \eta \eta }^{(k)}}+({f}_{\eta \eta \eta \eta }^{(k+1)}-{f}_{\eta \eta \eta \eta }^{(k)})\frac{\partial \varphi }{\partial {f}_{\eta \eta \eta \eta }^{(k)}}=0,\\ (1+{C}_{1}){f}_{\eta \eta \eta \eta }^{(k+1)}+\left\{(1+{C}_{1})\frac{2}{\eta +k}+\frac{k}{\eta +k}{f}^{(k)}\right\}{f}_{\eta \eta \eta }^{(k+1)}+\left\{\begin{array}{c}-(1+{C}_{1})\frac{1}{{(\eta +k)}^{2}}-\frac{k}{\eta +k}{f}_{\eta }^{(k)}\\ +\frac{k}{{(\eta +k)}^{2}}{f}^{(k)}-{M}^{2}\end{array}\right\}\,{f}_{\eta \eta }^{(k+1)}\\ +\left\{-\frac{k}{\eta +k}{f}_{\eta \eta }^{(k)}-\frac{2k}{{(\eta +k)}^{2}}{f}_{\eta }^{(k)}-\frac{k}{{(\eta +k)}^{3}}{f}^{(k)}-\frac{{M}^{2}}{\eta +k}+(1+{C}_{1})\frac{1}{{(\eta +k)}^{3}}\right\}{f}_{\eta }^{(k+1)}\\ +\left\{\frac{k}{\eta +k}{f}_{\eta \eta \eta }^{(k)}+\frac{k}{{(\eta +k)}^{2}}{f}_{\eta \eta }^{(k)}-\frac{k}{{(\eta +k)}^{3}}{f}_{\eta }^{(k)}\right\}{f}^{(k+1)}\\ ={C}_{1}\left({g}_{\eta \eta }^{(k)}+\frac{{g}_{\eta }^{(k)}}{\eta +k}\right)+\frac{k}{{(\eta +k)}^{2}}\{{f}^{(k)}{f}_{\eta \eta }^{(k)}-{({f}_{\eta }^{(k)})}^{2}\}-\frac{k}{{(\eta +k)}^{3}}{f}^{(k)}{f}_{\eta }^{(k)}+\frac{k}{\eta +k}\{{f}_{\eta \eta \eta }^{(k)}{f}^{(k)}-{f}_{\eta }^{(k)}{f}_{\eta \eta }^{(k)}\}.\end{array}\}$$

Presently (17) provides an arrangement of linear ODEs, where ${f}^{k}$ implies ${k}^{th}$ equation solution. For solution of linear ODEs, we swap derivatives with central differences that generate sequence $\{{f}^{(k)}\}$, created through subsequent linear system:

18 $$B{f}^{(k+1)}=C,\,B\equiv {B}_{n\times n}({f}^{(k)}),\,C\equiv {C}_{n\times 1}({f}^{(k)}),$$

where *n* shows numbering for grid points. Also Eqs. (12–14) are linear in *g*, $\theta$ and $\phi$ and therefore, sequences $\{{g}^{(k)}\}$, $\{{\theta }^{(k)}\}$ and $\{{\phi }^{(k)}\}$ are developed as:

19 $$\{\begin{array}{c}\left(1+\frac{{C}_{1}}{2}\right)\left({g}_{\eta \eta }^{(k+1)}+\frac{1}{\eta +k}{g}_{\eta }^{(k+1)}\right)+\frac{k}{\eta +k}{f}^{(k+1)}\,{g}_{\eta }^{(k+1)}-\frac{k}{\eta +k}{f}_{\eta }^{(k+1)}\,{g}^{(k+1)}\\ -{C}_{1}\left(2\,{g}^{(k+1)}+{f}_{\eta \eta }^{(k+1)}+\frac{{f}_{\eta }^{(k+1)}}{\eta +k}\right)=0\end{array}$$

20 $$\{{\theta }_{\eta \eta }^{(k+1)}+\frac{1}{\eta +k}(1+k\,Pr\,{f}^{(k+1)}){\theta }_{\eta }^{(k+1)}=0$$

21 $$\{\frac{1}{Sc}\,{\phi }_{\eta \eta }^{(k+1)}+\frac{1}{\eta +k}\left(\frac{1}{Sc}+k\,{f}^{(k+1)}\right){\phi }_{\eta }^{(k+1)}=0$$

where ${f}^{(k+1)}$ is taken into consideration to be eminent in above mentioned equations and their derivatives are estimated via central differences. The system of linear Eqs. (18–21) which are obtained after using the central differences are thus solved iteratively by SOR method.

## Results and discussion

Different physical parameters involving into fluid flow, microrotation, heat and mass are *M*, *Sc*, Pr, *k* and $\frac{\kappa }{\mu }={C}_{1}$. It is, therefore, important to investigate their effects on the velocities $f(\eta )\,\& \,f{\prime} (\eta )$, micro-rotation $g(\eta )$, temperature distribution $\theta (\eta )$ and concentration $\phi (\eta )$ (by varying each of them when the others are kept constant).

**Figure 1:**
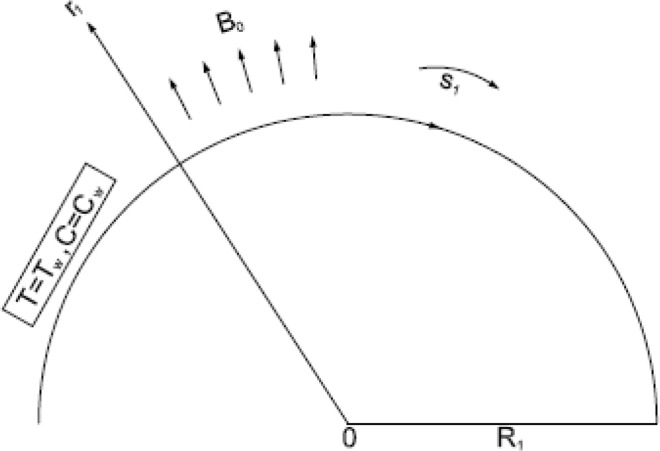
Geometry of the curved surface.

**Figure 2:**
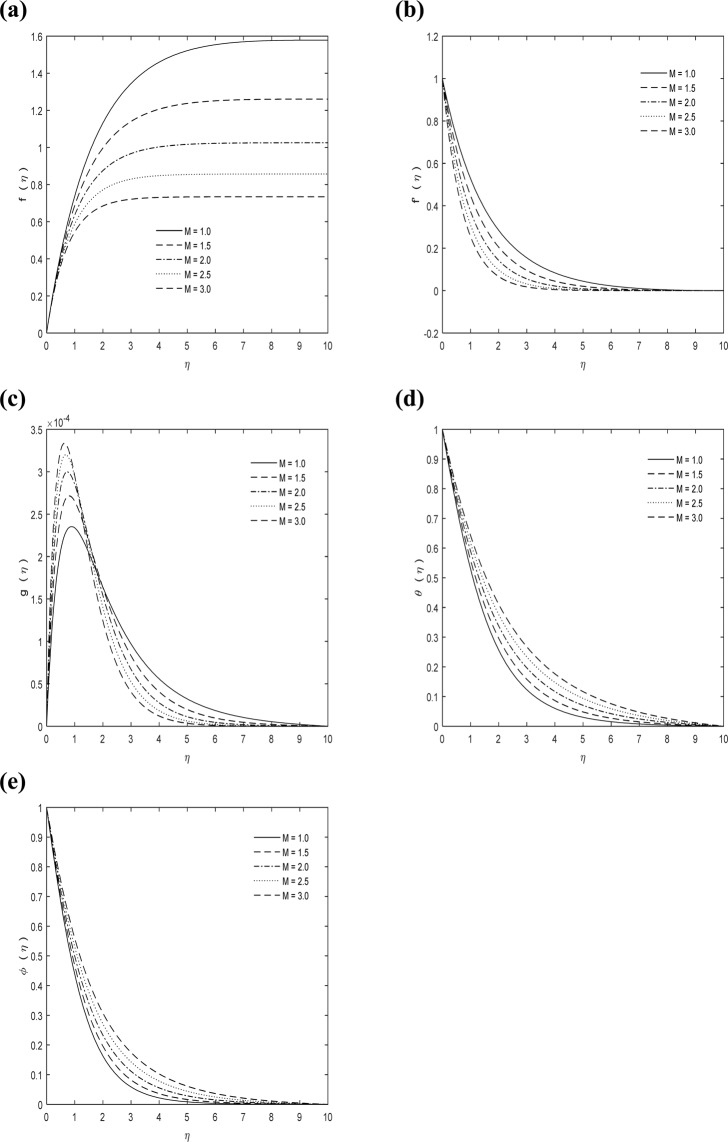
*M* Impact on **(a)** $f(\eta )$ **(b)** $f{\prime} (\eta )$ **(c)** $g(\eta )$ **(d)** $\theta (\eta )$ **(e)** $\phi (\eta )$ with $\kappa =7,\,{C}_{1}=5,\,\Pr =0.7,\,Sc=1$.

Figure 1 shows the physical model. Figure 2(a–e) exhibit impact of *M* on dimensionless velocity, micro-rotation, concentration and temperature fields. With increase in *M*, i.e. proportion of electromagnetic force to inertial force, velocity field diminishes. The Lorentz force is regarded in hydromagnetic flow because of existence of magnetic field. As *M* increases retarding force will increase and therefore velocity decreases. It may be seen in Figs. a and b. Also resolute that at any position on boundary layer temperature field add up with increasing *M*. The reason behind this is the use of a magnetic field on flow area that creates a Lorentz’s force, which takes action like retarding force and as a result temperature of fluid inside boundary layer increases as proven in Fig. d. Moreover, surface temperature of the sheet could be managed by controlling power of applied magnetic field. From Figs. c, e, it is observed that magnetic parameter diminishes micro-rotation profiles and increases concentration field. Due to its significant importance in industrial innovation, MHD study has extensive interest for the specialized technical fields and have applications in nuclear cooling reactors, biological transportation, drying processes, high temperature plasmas, astronomy, geophysics, sensors etc. Moreover, it is also important in earthquake assumption and MHD power generation. It is not only used in commercial power generation but also in hydroelectric power plants and nuclear power plants etc.

**Figure 3:**
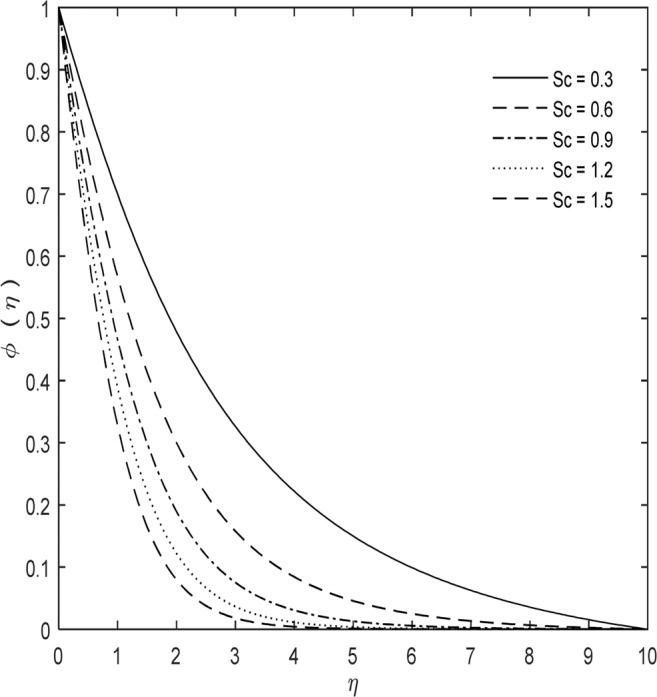
*Sc* impact on $\phi (\eta )$ with $\kappa =7,\,M=1,\,{C}_{1}=5$ and $\Pr =0.7$.

The impact of *Sc* on mass distribution is significant with increase in Schmidt number as shown in Fig. 3. The mass momentum transfer is described by Schmidt number. It is a dimensionless number and explained as ratio of kinematic viscosity to mass diffusivity and is characterized to use in fluid flows where simultaneous mass and momentum diffusion convection procedures take place.

Mass diffusivity is proportionality constant between molar motion because of molecular diffusion and inclination in grouping of species (or main impetus for diffusion). Mass diffusion in fluids increases with temperature, on the other hand *Sc* $(Sc=\upsilon /D)$, start decreasing with temperature if viscosity variation is inversely proportional to temperature. Physically *Sc* connects the thickness of mass transfer and hydrodynamic boundary layers. In heat transfer systems Schmidt and Prandtl numbers are correlated with each other.

*Sc* is a powerful tool for thermal and chemical engineers. It is basic widget in evolution of developed gas turbine combustors. Suitable temperature prognosis at exit and combustor wall plays a crucial role for gas turbine engineers because temperature profiles may diminish the lifetime of combustor and the turbine connected behind it. *Sc* is also important for numerical simulations. So because of this it has been applied to numerous numerical studies, like jet flows.

**Figure 4:**
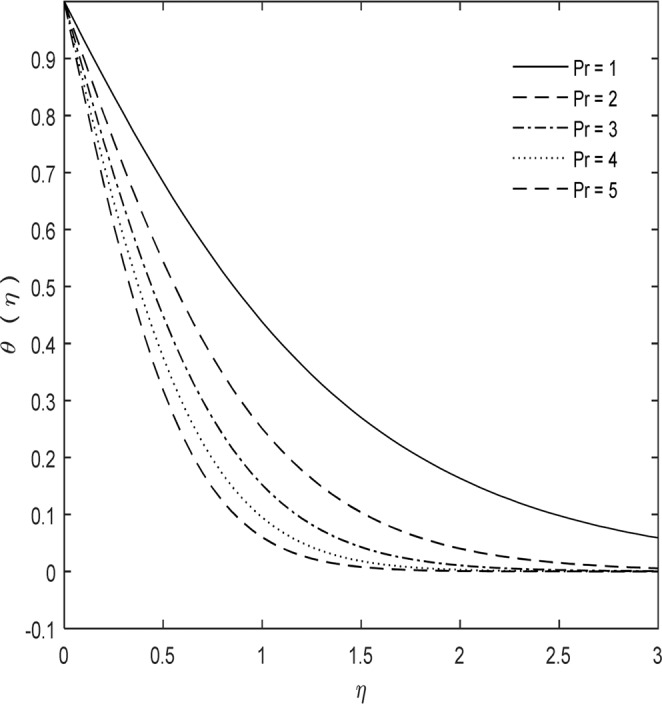
Pr impact on $\theta (\eta )$ with $\kappa =7,\,M=1,\,{C}_{1}=5$ and $Sc=1$.

Figure 4 demonstrates influence in temperature distribution θ(η) for various values of Pr. It is established from Fig. 4 that temperature reduces by escalating the Pr. The Pr is based on ratio of momentum diffusion to thermal diffusion. By increase Pr thermal diffusion reduces and so thermal boundary-layer turns out to be thinner. Pr gives information about fluid type. Additionally it gives data about thickness of hydrodynamic and thermal boundary layer. Relative thickness of momentum and thermal boundary layers may be controlled by Prandtl number in heat transfer systems. This number is also important for different properties like thermal conductivity and viscosity. Thermal diffusivity monopolizes for smaller Prandtl values (Pr ≪ 1), on the other hand momentum diffusivity dominates for larger Prandtl values (Pr ≫ 1). The Pr number implies numerous computations of heat transfer in fluid metal reactors. In Generation IV, reactors liquid metals are used as reactor coolants like lead and sodium cooled fast reactor.

**Figure 5:**
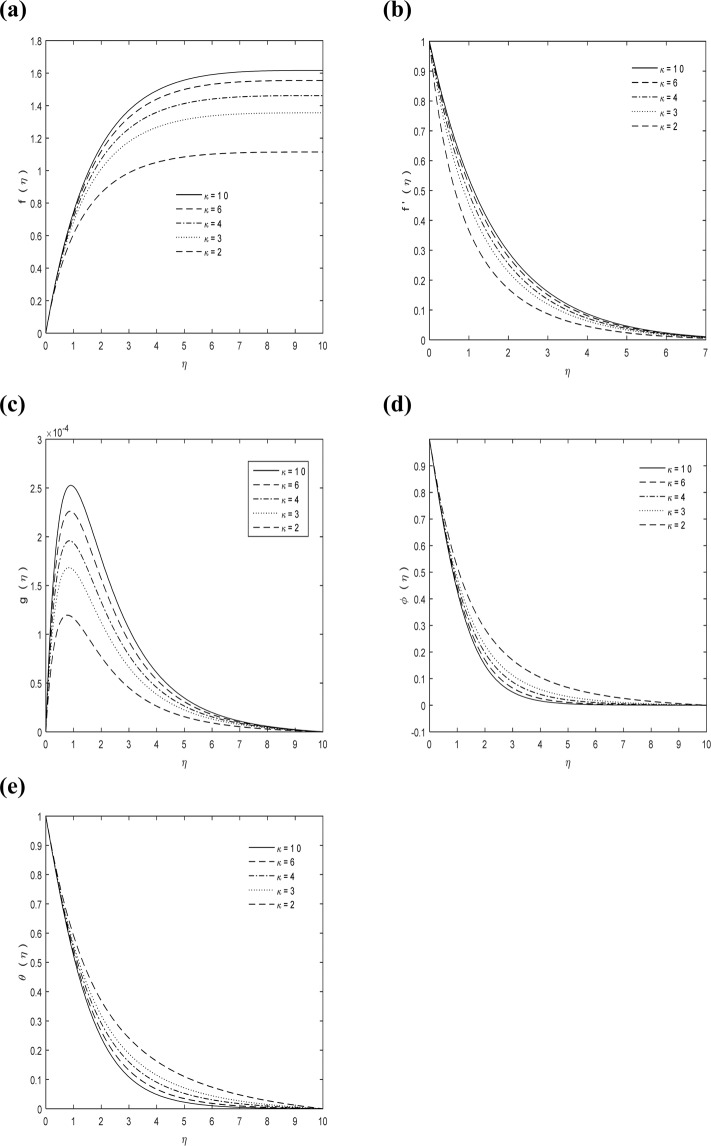
*k* impact on **(a)** $f(\eta )$ **(b)** $f{\prime} (\eta )$ **(c)** $g(\eta )$ **(d)** $\phi (\eta )$ **(e)** $\theta (\eta )$ with $M=1,\,{C}_{1}=5,\,\Pr =0.7,\,Sc=1$.

From Fig. 5(a–c) it can be noticed that the values of *k* decline the velocities and micro-rotation whereas both concentration and temperature of fluid are increasing as in Fig. 5(d,e). Curvature reciprocal is known as radius of curvature. For curve it is equal to circular arc radius which best fits curve at this point. For surfaces circle radius is called radius of curvature and it is changed as we move alongside the curve. Radius of curvature is also important in physics because it plays a major role in optical designing of spherical lenses and mirrors whereas it is also important in mathematics because it is used in differential geometry as Cesaro equation or as three part equation for beams bending.

When train tracks are designed by engineers, they make sure that track curvature will be secure, safe and provide comfort ride for given velocity of trains. It is also used by civil engineers in designing of highways and extremely sharp turns of principle roads. One desires large radii of curvature on high speed roadways so drivers may sustain control. Governments may also specify minimal curvature radius in such situations. In traffic circles, radius also becomes a road speed limit function.

**Figure 6:**
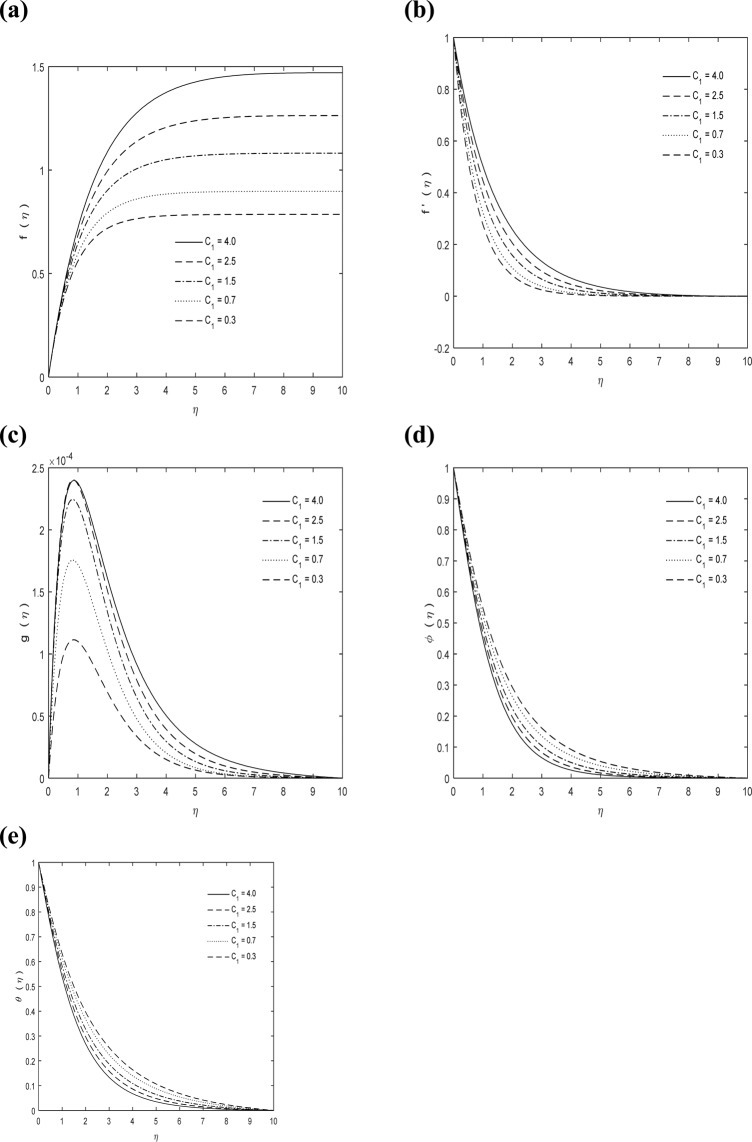
*C*_1_ impact on **(a)** $f(\eta )$ **(b)** $f{\prime} (\eta )$ **(c)** $g(\eta )$ **(d)** $\phi (\eta )$ **(e)** $\theta (\eta )$ with $\kappa =7,\,M=1,\,\Pr =0.7$ and $Sc=1$.

The same behavior is observed in material parameter *C*_1_ as delineated in Fig. 6(a–e). The *C*_1_ is dimensionless number and explained as ratio of vortex viscosity to dynamic viscosity. In deviatoric stress tensor modeling material parameter (vortex viscosity) performs essential function; it may not be possible to ignore temperature impact on the micropolar fluid dynamic viscosity. Vortex viscosity performs a crucial role in magnetic field dynamics and is used in technological important materials for different applications. The stress antisymmetric portion is quantified through vortex viscosity.

## Conclusions

In present study, it has been noted that magnetic parameter tries to straighten concentration profiles. The influence of escalating values of radius of curvature is to put down velocity field while temperature is enhancing with increasing *k*. Pr causes to increase cooling rate in conducting flows. It is noticed that external magnetic field tends to raise temperature profiles.
